# The O3 guidelines: open data, open code, and open infrastructure for sustainable curated scientific resources

**DOI:** 10.1038/s41597-024-03406-w

**Published:** 2024-05-29

**Authors:** Charles Tapley Hoyt, Benjamin M. Gyori

**Affiliations:** 1 Khoury College of Computer Sciences, Northeastern University, Boston, MA USA; 2 Department of Bioengineering, College of Engineering, Northeastern University, Boston, MA USA

**Keywords:** Databases, Standards, Software, Data publication and archiving, Databases

## Abstract

Curated resources that support scientific research often go out of date or become inaccessible. This can happen for several reasons including lack of continuing funding, the departure of key personnel, or changes in institutional priorities. We introduce the Open Data, Open Code, Open Infrastructure (O3) Guidelines as an actionable road map to creating and maintaining resources that are less susceptible to such external factors and can continue to be used and maintained by the community that they serve.

## Background & Summary

Scientific discovery crucially relies on resources that combine expert-curated data with surrounding software code and services. For example in the life sciences, these include model organism databases, pathway databases, ontologies, and many other curated resources. Some well-known and widely used resources include the Mouse Genome Database^1^, the Gene Ontology^2^, the Disease Ontology^3^, WikiPathways^4^, and Wikidata^5^. Despite their utility to the community, too often, resources go out of date, are abandoned, or become inaccessible. The pervasiveness of this problem can be estimated by checking for accessibility of curated resources cataloged in the Bioregistry^6^: the Bioregistry Health Report (https://biopragmatics.github.io/bioregistry/health) finds that only 1,121 of 1,477 (75%) of cataloged resources are accessible as of October 2023^7^. Most curated resources would also benefit from regular updates, but often, despite remaining accessible online, their content ceases to be maintained. For example, the NCI Pathway Interaction Database^8^ remains frequently reused despite being retired in 2016. Reasons for resources becoming inaccessible or obsolete may include the fact that maintenance is susceptible to fluctuations in funding, personnel, and institutional priorities. To overcome these issues, we need better technical and social processes for creating resources that are less susceptible to such external factors and can continue to be used and maintained by the communities that they serve. We consider resources with these qualities *sustainable*.

**Fig. 1:**
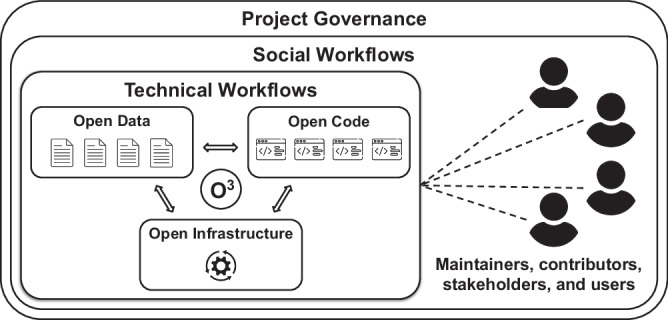
A schematic diagram of how social workflows, technical workflows, and project governance interact with open data, open code, and open infrastructure (O3).

Here, we introduce the Open Data, Open Code, and Open Infrastructure (O3) Guidelines for the creation and maintenance of curated resources which promote sustainability through a combination of technical workflows, social workflows, and progressive governance models. Together, these support and encourage community-facing curation (Fig. 1). In summary, (1) the *technical* aspect of O3 necessitates using open data, open code, and open infrastructure. Both data and code are permissively licensed and kept together under public version control. This enables anyone to directly suggest improvements and updates. Further, it recommends using hardware and software infrastructure that supports automation in response to various actions performed by contributors and maintainers. For example, this includes running quality assurance workflows in response to new contributions and the generation of exports in multiple formats in response to running a release workflow. (2) The *social* aspect of O3 prescribes the composition of training material, curation guidelines, contribution guidelines, and a community code of conduct that encourage and support potential community curators. It requires the use of public tools for suggestions, questions, discussion as well as social workflows for the submission and review of changes. (3) The *governance* aspect of O3 necessitates the division of responsibilities and authority across multiple institutions, making the resource more robust to fluctuation in funding and personnel, such as when reviewing and applying changes to the data or code. O3 prescribes liberal attribution and acknowledgment of the individuals and institutions, both internal and external to the project, who contribute on a variety of levels such as to data, code, discussion, and funding. More generally, the O3 Guidelines suggest that a minimal governance model be codified and instituted as early as possible in a project’s lifetime.

In what follows, we describe the O3 Guidelines in detail (illustrated through resources well known in the biomedical community) and provide a practical path to creating new sustainable resources as well as revitalizing existing ones.

## O3 Guidelines

We provide an actionable, step-by-step guide to O3 that covers each of the technical, social, and governance aspects of a sustainable project.

### Version control your data and code

A version control system is a tool that tracks changes to files and also enables multiple users to work on the same files concurrently and asynchronously, then mediate resolving conflicts if they arise. The most popular version control system is *git* (https://git-scm.com). Web-based collaborative services such as GitHub (https://github.com) are built on top of Git and enable interaction with the version control system through a web interface, a desktop graphical user interface, or the command line. While version control systems have traditionally been used for software code, curated resources that use version control systems for both their data and code together are more organized, accessible, engaging, and easier to maintain. Version control services secondarily act as a way of distributing data and code in an open way. This greatly improves on classical approaches to maintaining and distributing resources on *ad hoc* infrastructure such as university FTP servers, which are more susceptible to becoming inaccessible following changes in the funding or employment of the group that created them. Having recognized these advantages, resources such as the Gene Ontology^2^ and most OBO Foundry ontologies are curated on GitHub.

Version control systems have some limitations: they are optimized for text-based files, and therefore work inefficiently for very large data files as well as files that are in complex formats (e.g., PowerPoint presentation) or binary formats (e.g., compressed data). However, there are several data repositories that serve as alternatives to version control systems such as Zenodo (https://zenodo.org), Dryad (https://datadryad.org), DataCite (https://datacite.org/), and FigShare (https://figshare.com) for storing and sharing such files. These repositories provide less granular version control and contribution modes than GitHub but have the advantage of maintaining formally registered metadata for a resource.

### Permissively license your data and code

A license is a statement about how data and code in a resource can be used, modified, and redistributed. Using a recognizable, permissive license such as the Creative Commons (CC) licenses CC BY or CC0, as suggested by Carbon *et al*.^9^, not only encourages contribution but also promotes sustainiability as the content can be incorporated into a successor resource in case the original is discontinued or abandoned. For example, the LOTUS natural product database^10^ incorporated and extended several abandoned chemical information resources (see Appendix 1 in^10^).

Resources using less permissive licenses for data can have several issues with sustainability. Licenses that explicitly restrict modification, such as the CC BY-ND license, do not promote sustainability as the licensed content can not be reformed, extended, or easily incorporated into a successor resource. Similarly, licenses that apply copyleft restrictions, such as the CC BY-SA license, do not promote sustainability as content licensed under their terms can not be incorporated into resources with more permissive licenses. Licenses that use non-standard language increase the burden on potential users or contributors to understand implications on reuse and are therefore discouraged. Potential contributors who want to maximize the reusability of their contributions might be less inclined to contribute to resources with any of the previously mentioned licensing restrictions. One concern about permissive licenses is that they in principle allow for credit not to be properly given to the resource. Carbon *et al*. suggest this is not the case in practice^9^. For example, the Disease Ontology switched from the CC BY to the more permissive CC0 license and nevertheless reports a high citation rate by its users^3^. We note that commercial resources follow an alternative sustainability model to O3. Instead, they use income from sales to pay for maintenance and therefore often adopt restrictive licenses that prevent redistribution or reuse.

In addition to data licensing, licensing considerations are also important for software code used in a curated resource. Using an OSI software license (https://opensource.org/license) that is familiar to the community and doesn’t carry commercial restrictions is most supportive of reusability and sustainability. Best practices for research software as introduced in the FAIR4RS Principles^11^ include assigning persistent identifiers, providing high quality metadata, and using standard interfaces and exchange formats.

Finally, a project should clearly communicate how to acknowledge and cite its data and code. This can be achieved, for instance, by obtaining a DOI (Digital Object Identifier) from Zenodo, FigShare or DataCite, independent of the availability of a peer-reviewed scientific publication describing the resource. In addition to providing a basis for citation, this promotes findability as an aspect of FAIR data^12^. For smaller projects that do not lend themselves to full research articles, several journals have “data note” formats for short, concise articles describing data.

### Make your data maintainer-friendly and user-friendly

Projects that make their curated data user-friendly are better able to elicit community contribution, train maintainers, and reduce the cognitive burden for each. We suggest three avenues toward this goal.

First, this can be accomplished by storing data in a simple, concise, and non-proprietary file format. The ideal file format should be easily editable by both humans and machines, compatible with version control systems’ tools for visualizing changes (often called *diffs*), and displayable by popular hosting services like GitHub. JSON, TSV, and YAML are examples of formats that meet these criteria. Further, the format should not be more verbose than what is necessary for the curation goals of the project. For instance, XML—while also meeting the above criteria—may not be user-friendly due to its verbosity which makes it less approachable for human editing. It is further desirable for data to be “canonicalized” in a way that avoids unnecessary changes when edited by tools.

Second, projects that reuse external standards for data modeling are more approachable than those that use bespoke internal standards. A resource reusing an external schema often benefits from the existence of associated documentation, tutorials, and tooling. For example, a protein-protein interaction database can be curated using the PSI-MITAB^13^ schema within the TSV file format giving access to detailed reference material and software packages for working with the data. Similarly, groups of similar resources can share standard semantics and schemas for files in their repositories, a good example being the ecosystem of OBO Foundry Ontologies^14^.

More fundamentally, it is preferable to adopt external controlled vocabularies or ontologies for annotating data, models, and knowledge. In case existing controlled vocabularies are insufficient, the preferred approach is to work with the maintainers of such resources to make improvements. Similarly, using generic community standards for identifying concepts appearing in the resource reduces the cognitive burden on potential external contributors as well as on users. This can be accomplished at the syntactical level by using URIs or compact URIs (CURIEs) for referencing biomedical concepts and at the semantic level by using standardized CURIE prefixes, aligned to a registry like the Bioregistry^6^.

Third, projects that only maintain a single *editable* instance of their data (i.e., a single source of truth) are easier to modify. Such projects can more naturally avoid data duplication and eliminate the need to make the same changes in multiple places, which is often error-prone and can result in inconsistencies. For example, the Bioregistry^6^ stores its data in a single JSON file that is documented by an accompanying JSON schema. Similarly, the Ontology Development Kit (ODK)^15^ enables components of ontologies to be curated in TSV templates as opposed to the more complicated serializations of OWL files. While maintaining a single editable instance of each data file, projects that need to distribute their data in multiple formats can follow the suggestions in the “Technical Workflows” section below instead of manually maintaining duplicate copies of the same data.

### Use technical workflows for automation

Projects that leverage open infrastructure (hardware and software resources available via GitHub or other cloud providers) and automation are able to better assist contributors, reduce maintainer effort, and ultimately, benefit consumers. There are four key areas where automation benefits curated resources: quality control, generation of artifacts, releases, and deployment.

Automating quality control reduces the burden on maintainers for reviewing contributions while improving the experience for contributors by giving feedback more quickly and in an impartial way. Modern version control platforms like GitHub offer continuous integration (CI) workflows that are triggered automatically on all changes or commits. This can be used to implement quality control checks for both data and code. Specific CI workflows may include checking data for the correct formatting and semantics, such as asserting that entries in a column of a spreadsheet describing proteins are written with identifiers from UniProt and validated using a certain regular expression. Such quality control checks are valuable for projects with many contributors by providing an objective, deterministic way of communicating issues to contributors that should be handled before a maintainer makes a review. As an example, Biomappings^16^ uses CI to check that its semantic mappings use standardized CURIEs to reference the subject, predicate, object, and other metadata for each record. A similar process can be applied to code to check for code style, the completeness of documentation, and test coverage.

Automation can reduce the technical experience and time investment required of maintainers to generate derived artifacts such as charts, tables and other summaries of a resource. For example, the TIWID database^17^ automatically generates a collated data export and generates summary charts any time changes to its underlying data are pushed. Automation further supports the previously mentioned concept of the single source of truth by allowing for the generation of derived views. For example, a resource that is curated in JSON as the single source of truth can be projected and exported into a simplified tabular format, or enriched with additional semantics to be exported into a linked data format such as RDF.

In addition to tracking changes through a version control system, many projects make releases with explicit version numbers. Automation can be used to facilitate this such as through git’s tagging system and GitHub’s release system. This can be further integrated with an external archival system such as Zenodo to provide long term storage and persistent identifiers for each release. The ODK has been highly influential in automating the release process for OBO Foundry ontologies, an approach that has reduced barriers for reuse and interoperability.

Automation is also useful for packaging and deploying the data and code for a project. For example, this can involve wrapping the curated data in a Python package which exposes the data through a programmatic API. Packaged data and code can be containerized with technologies such as Docker. This makes packages more portable and allows for deployment to a cloud provider such as Amazon Web Services (AWS) on a fit-to-purpose machine. For example, the Bioregistry Python package is only a few megabytes, is containerized in a Docker image that is less than 100 megabytes, and can run on the smallest available AWS instance type which costs less than 30 USD per year. Projects that only need a simple website can use a simple static site generator and a templating language. For example, GitHub provides the Jekyll environment with the Liquid templating language that can deploy directly from content inside the repository storing the data and code.

### Use social workflows for collaboration

Social workflows enable a project’s community of maintainers, contributors, and users to more effectively communicate and collaborate. We highlight social workflows enabling data- and code-level discussion, contribution, review, and project-level discussion.

Modern version control platforms like GitHub implement a variety of features that facilitate social workflows^18^. Namely, each project comes with an issue tracker and discussion board for transparent community engagement. These systems also allow for external contributions to be suggested, reviewed, and discussed in a transparent fashion. Automated quality assurance and other CI workflows are often integrated into the contribution process to automatically and objectively enforce pre-defined data and code standards. Projects that have not yet adopted such workflows often have difficulty on-boarding contributors and review external contributions less efficiently.

Complementary discussion platforms including instant messaging (e.g., on Slack) or forums (e.g., Google Groups) are often used to allow discussion outside the public version control platform. Successful projects encourage archived, transparent discussion that is searchable and gives important context to new contributors and users. Email lists are another option, but some have the caveat that their history is stored within email accounts, which only covers from when a member joins to when they lose email access (e.g., if they move institutions). Private email discussions have the same caveats and are not publicly accessible, so projects should highly discourage contributions or maintenance through this kind of channel.

### Establish clear project governance

The goal of establishing project governance is to communicate the expectations on how contributors, maintainers, users, and stakeholders should act and how the project should be maintained over time. Clear and transparent project governance, even if minimal, can help build trust in the project and its ability to evolve over time.

For projects combining open data, open code, and open infrastructure, governance is important for defining the roles and responsibilities for project members. Most importantly, this includes defining a code of conduct. The Contributor Covenant (https://www.contributor-covenant.org) is a good starting point for most projects. It essentially states that maintainers, contributors, users, or any other participants in the community should be kind and courteous to each other. Next, the code of conduct defines the responsibilities of administrators and a set of standard operating procedures (SOPs) for how one becomes an administrator and how one leaves (or is removed from) the role. For instance, while exiting a position is typical throughout a research career, it is important to also prepare for the possibility that the community needs to remove an individual. This process is more transparent and objective when there are SOPs to refer to on why and how this must occur. Similar SOPs should be established for other members, such as who has rights to make reviews, accept changes, or decide on updates to data models.

Governance should make expectations clear about who will be included as co-authors on publications. An ideal SOP states that all material contributors and meaningful contributors to discussions are automatically eligible to be co-authors. Further, journals are increasingly adopting the Contributor Roles Taxonomy (CRediT) as a tool to enable annotating additional context for the contribution type and impact of each co-author^19^. It remains an open discussion how to develop fair yet flexible strategies for determining the (shared) first author position, the (shared) corresponding author position, and how to determine who is responsible for paying article processing charges (APCs) for publications.

Finally, it is likely that adjustments to the governance model have to be made over time to accommodate the evolving needs of the project. Therefore, SOPs for making changes to the governance model itself should also be included.

### Attract and engage contributors

Contribution guidelines communicate what types of contributions can be made to the project and describe the procedure for contributions. They often include examples and tutorials that help users understand the scientific purpose of the resource as well as any tools that are needed to contribute. Projects that offer many tracks toward contribution are often able to recruit more contributors and therefore improve their sustainability. These can be through many avenues, such as submitting an issue via an issue tracker, improving documentation, or making material contributions to data or code. For example, WikiPathways has a very detailed set of contribution guidelines and tutorials and has been successful in engaging a large number of contributors.

Giving credit early and often provides an incentive for contributors, maintainers, and users to actively participate. Credit can be given to individuals through multiple avenues. If a contributor is named in a git commit, the contributor appears in the history of changes to the resource as well as the list of contributors. Attribution can also make use of ORCiD identifiers inside the data model to attribute specific contributions. Finally, attribution can happen through offering co-authorship on scientific publications about the resource as described before. In addition to individual contributors, credit can be given at the institutional level or to funding sources by including logos of prominent contributors’ institutions as well as by listing contributors’ funding statements for their grants prominently. We refer to the OBO Academy’s tutorial for additional information (https://oboacademy.github.io/obook/howto/open-science-engineer).

Where a project is maintained may also affect its ability to attract and engage contributors. For example, community contributors are less likely to feel ownership over a project that is hosted in a place specific for a single organization or heavily branded with references to a single organization. This can decrease contributor engagement. Instead, it is preferable to host projects in a neutral version control system, such as GitHub, instead of an internal version control system. Further, it is preferable to host projects in a project-specific space instead of an institution or individual’s space within the version control system. This has the additional benefits that maintainers can more easily stay involved, even if they move institutions.

## Discussion

Here, we introduced the O3 Guidelines and proposed a combination of version control, permissive licenses, data standardization, technical workflows, social workflows, project governance, and community engagement as key toward creating sustainable resources. Below, we discuss the relationship between the O3 Guidelines and related standards, the historical context that enabled the development of the O3 Guidelines, their limitations, and the potential impact of their adoption.

### Relationship to other standards

We see the O3 Guidelines as an addition to the set of tools researchers and funding agencies have for creating new resources that meet requirements of openness and sustainability. The O3 Guidelines can also be operationalized to assess the quality and utility of existing resources. Notably, O3 is complementary to the FAIR data principles^12^, which focus on the quality of metadata and documentation of data and code, whereas the O3 Guidelines provide an actionable road map toward sustainability. O3 is related to, and expands upon the open data and open code practices suggested by the Blue Obelisk project^20^ and the usage of permissive licenses prescribed by Open Data practices^21^ by further guiding the creation of new resources to organize its data and code in a way that promotes community engagement, and therefore sustainability. Similarly, O3 complements the TRUST Principles^22^ by providing actionable steps toward its nominal goals of transparency, responsibility, user focus, sustainability, and technological capabilities. Again, O3 takes inspiration in many places from the OBO Foundry Principles^14^. The O3 guidelines are also synergistic with organizations that aim to improve sustainability across biomedical resources such as ELIXIR^23^ or the Global Biodata Coalition^24^ by providing centralized infrastructure. Adopting O3 will help integrate individual resources with such organizations, while also being applicable to how the development of centralized infrastructure approaches sustainability.

### Historical context

Many of the tools and resources referenced in the O3 Guidelines have only become broadly available to a general audience in recent years. This includes free continuous integration and continuous delivery systems that are integrated with GitHub. More generally, version control systems with integrated social tools have only recently begun to be adopted by researchers, and discovering the most effective ways to use them is an ongoing process. These are part of a longer-term trend toward harnessing the potential of technology to improve the scientific process.

### Limitations

Wider adoption of the O3 Guidelines presents several challenges. Because many of the tools and software implementing the technical and social workflows described above are relatively new, some developers and maintainers of curated resources might not yet have experience with them and will therefore require training. This can be mitigated by providing additional training materials, hosting workshops, and other outreach following a model that has been demonstrated to be successful by the OBO Academy^25^.

Maintainers of curated resources may face challenges when considering implementing the O3 Guidelines both in terms of allocating effort and funding. This presents an opportunity for scientific software developers to support the transition. Further, it motivates incorporating more explicit provisions for technical maintenance in new grants. Organizations like the Global Biodata Coalition and Research Software Alliance are promoting discourse around progressive funding models that may fill this currently unaddressed need. Further, we envision that funding agencies will be able to use the O3 Guidelines as a tool for helping grant applicants conceive better data management and sustainability plans as well as for more actively encouraging the adoption of the O3 Guidelines in order to increase their return on investment.

As we alluded to above, one technical limitation of the O3 Guidelines is using version control systems to maintain large data files. To overcome this, where necessary, curated resources can be broken down into smaller, self-contained files within a given version controlled repository that may themselves be appropriate for maintenance under an O3 model. For example, UniProt^26^ maintains several ontologies for diseases, cell types, and other controlled vocabularies that could each be curated as a stand-alone file. Similarly, UniProt contains many facets for each protein that could be curated as individual resources (in fact, in many cases, UniProt already benefits from importing existing fit-for-purpose resources).

Finally, we foresee challenges in the implementation of the O3 Guidelines, and more generally in the shift toward community-driven projects, due to conflicts with the existing incentive structure for individual scientists, institutions, and funding bodies^27^. Under the current model, researchers are incentivized to build their own resources in order to publish them as the primary or senior author. While revising this incentive structure goes beyond the O3 Guidelines, we expect that the shift toward more granular and more liberal attribution prescribed by the O3 Guidelines will enable new ways of thinking about these incentives.

### Potential and impact

We believe that the adoption of the O3 Guidelines has the potential to enact a long-term, widespread positive impact on research. A direct impact of adopting these guidelines is the community-based sustainability of curated scientific resources. However, there are a number of further benefits to users of such resources. First, fewer resources would be needed to re-implement similar databases or to do “digital archaeology” to identify and make use of abandoned databases. Lowering the bar for giving credit (e.g., on co-authorship) could also reduce the incentives for competitive, duplicate efforts, enabling the more efficient use of resources and funding. With the O3 Guidelines, a broader community has the opportunity to take part in data review, which is expected to ultimately lead to better quality data. Second, we see these guidelines as a path to democratizing science and making it more inclusive toward typically under-represented communities. For example, the sub-discipline of biocuration is biased toward a small number of organizations in North America, the UK, and Switzerland. When these organizations’ priorities shift (e.g., a database is abandoned), it has a global impact. If these organizations were to adopt the O3 Guidelines, it would increase sustainability and make it possible for individuals or groups from other regions to contribute.

## References

[1] Bult CJ (2019). Mouse Genome Database (MGD) 2019. Nucleic Acids Research.

[2] Consortium GO (2021). The Gene Ontology resource: enriching a GOld mine. Nucleic Acids Research.

[3] Schriml LM (2022). The Human Disease Ontology 2022 update. Nucleic acids research.

[4] Martens M (2021). WikiPathways: connecting communities. Nucleic Acids Research.

[5] Waagmeester, A. *et al*. Wikidata as a knowledge graph for the life sciences. *eLife* **9**, 10.7554/eLife.52614 (2020). 10.7554/eLife.52614 PMC7077981 32180547

[6] Hoyt CT (2022). Unifying the identification of biomedical entities with the Bioregistry. Scientific Data.

[7] Hoyt CT (2023). Zenodo.

[8] Schaefer CF (2009). PID: the Pathway Interaction Database. Nucleic Acids Research.

[9] Carbon S (2019). An analysis and metric of reusable data licensing practices for biomedical resources. PloS one.

[10] Rutz A (2022). The LOTUS initiative for open knowledge management in natural products research. eLife.

[11] Barker M (2022). Introducing the FAIR Principles for research software. Scientific Data.

[12] Wilkinson MD (2016). The FAIR Guiding Principles for scientific data management and stewardship. Scientific Data.

[13] Perfetto L (2019). CausalTAB: the PSI-MITAB 2.8 updated format for signalling data representation and dissemination. Bioinformatics.

[14] Jackson RC (2021). OBO Foundry in 2021: Operationalizing Open Data Principles to Evaluate Ontologies. Database.

[15] Matentzoglu, N. *et al*. Ontology Development Kit: a toolkit for building, maintaining and standardizing biomedical ontologies. *Database* **2022**, 10.1093/database/baac087 (2022). 10.1093/database/baac087 PMC9547537 36208225

[16] Hoyt, C. T., Hoyt, A. L. & Gyori, B. M. Prediction and curation of missing biomedical identifier mappings with Biomappings. *Bioinformatics* **39**, 10.1093/bioinformatics/btad130 (2023). 10.1093/bioinformatics/btad130 PMC10076045 36916735

[17] Willighagen E (2021). Zenodo.

[18] Perez-Riverol Y (2016). Ten simple rules for taking advantage of Git and GitHub. PLoS Computational Biology.

[19] McNutt MK (2018). Transparency in authors’ contributions and responsibilities to promote integrity in scientific publication. Proceedings of the National Academy of Sciences.

[20] O’Boyle NM (2011). Open data, open source and open standards in chemistry: The blue obelisk five years on. Journal of Cheminformatics.

[21] Jati PHP, Lin Y, Nodehi S, Cahyono DB, van Reisen M (2022). FAIR Versus Open Data: A Comparison of Objectives and Principles. Data Intelligence.

[22] Lin D (2020). The TRUST principles for digital repositories. Scientific Data.

[23] Harrow J (2021). ELIXIR: providing a sustainable infrastructure for life science data at European scale. Bioinformatics.

[24] Cook, C. & Cochrane, G. The Global Biodata Coalition: Towards a sustainable biodata infrastructure. *Biodiversity Information Science and Standards* **7**, 10.3897/biss.7.112303 (2023).

[25] Vasilevsky N (2022). Zenodo.

[26] The UniProt Consortium (2023). UniProt: the Universal Protein Knowledgebase in 2023. Nucleic Acids Research.

[27] Rawat S, Meena S (2014). Publish or perish: Where are we heading?. Journal of research in medical sciences: the official journal of Isfahan University of Medical Sciences.

